# Posture of Healthy Subjects Modulated by Transcutaneous Spinal Cord Stimulation

**DOI:** 10.3390/life13091909

**Published:** 2023-09-14

**Authors:** Natalia Shamantseva, Olga Timofeeva, Alisa Gvozdeva, Irina Andreeva, Tatiana Moshonkina

**Affiliations:** Pavlov Institute of Physiology, Russian Academy of Sciences, 199034 St. Petersburg, Russia; drolli@inbox.ru (O.T.); kukumalu@mail.ru (A.G.); ig-andreeva@mail.ru (I.A.); moshonkina@infran.ru (T.M.)

**Keywords:** postural control, spinal cord, transcutaneous electrical stimulation, healthy subjects, cognitive style, supporting leg

## Abstract

Transcutaneous electrical stimulation of the spinal cord is used to restore locomotion and body weight support in patients with severe motor disorders. We studied the effects of this non-invasive stimulation on postural control in healthy subjects. Stimulation at the L1–L2 vertebrae was performed to activate the extensor muscles of the lower limbs. Because postural regulation depends on the cognitive style, the effects of the stimulation were analyzed separately in field-dependent (FD) and field-independent (FI) participants. During the study, FD and FI participants (*N* = 16, 25 ± 5 years, all right dominant leg) stood on a force platform in a soundproof chamber with their eyes closed. Stimulation was applied in the midline between the L1 and L2 vertebrae or over the left or right dorsal roots of the spinal cord; under the control condition, there was no stimulation. Stimulation destabilized posture in healthy subjects, whereas patients with movement disorders usually showed an improvement in postural control. In the FD participants, left dorsal root and midline stimulation increased several postural parameters by up to 30%. Dorsal root stimulation on the side of the supporting leg reduced postural control, while stimulation on the side of the dominant leg did not. No significant changes were observed in the FI participants.

## 1. Introduction

Upright posture control, i.e., antigravity efforts combined with stabilization of the positions of body segments, is a complicated process. The spinal cord (SC) plays a crucial role in the system of antigravity balance regulation.

### 1.1. Spinal Cord Stimulation as a Tool to Study Postural Control

After the method of electrical stimulation of the SC began to be actively used in studies of locomotion and postural regulation [1], it became clear that much is still unknown about the involvement of spinal networks in postural control. In experiments on decerebrated cats, it was shown that their spinal networks could completely control their body weight support in the absence of supraspinal influences in the case of stimulation of the L5 spinal segment [2]. In the study, the SC was stimulated invasively by placing electrodes on the dura mater of the cord. Later, the technology of transcutaneous electrical stimulation (tES) of the human SC was invented [3]. In this method, active electrodes are placed on the skin of the back above the vertebrae. It is a non-invasive method of SC electrical stimulation. The results of a study on subjects who were unable to stand due to severe SC trauma showed that with tES of the SC at the level of the T11 or L1 vertebrae (above the L1–L4 spinal segments [4]), all patients (*N* = 15) could independently control their vertical posture with minimal external support from the knees or pelvis, and some of them (*N* = 7)—without any support [5]. Thus, in the absence of supraspinal influences, when the possibility of regulation by sensory systems and processes occurring in the brain is limited, activation of the spinal networks of the SC by stimulation of the L1–L4 spinal segments makes it possible to effectively control the vertical posture and support the body weight.

Normally, the maintenance of upright standing is a complex process that relies on the combined activation of different muscle groups. The use of tES of the SC in studies on the physiological mechanisms of postural maintenance has a significant advantage—this method allows for non-invasive activation of SC networks, including those in normal subjects, i.e., to study SC neural networks in humans. It has been demonstrated that non-invasive spinal stimulation can precisely modulate the activity not only of the spinal locomotor center but also of the motor pools of the muscles of the lower extremities [6]. For this reason, tES is particularly useful in the study of posture.

### 1.2. Different Roles of Flexors and Extensors in Postural Control

Human standing control solves two tasks simultaneously: one is to provide and distribute tonic muscle activity to stabilize body segments (“posture”), and the other is to neutralize internal or external perturbations of body segments (“equilibrium”) [7]. The “posture” task is mainly performed by the extensor muscles, which are mainly composed of slow tonic fibers, and the “equilibrium” task, such as locomotion, is mainly performed by the flexor muscles, which are mainly composed of fast fibers. Within the lumbar SC, the flexor and extensor nuclei are anatomically separated [4,8], allowing tES to be used for the targeted control of both flexors and extensors. Recent research has shown that tES at the L1–L2 vertebral level activates lower limb extensors during stepping [6]. In order to specifically target these muscle groups, active electrodes are placed to the side of the midline of the spinal cord above the dorsal roots. Thus, tES at the L1–L2 level may also affect vertical postural stability via changes in the tonic extensor activity.

### 1.3. Cognitive Style Influences Coordination of Body Segments in Postural Control

The stability of an individual’s upright standing relies on the complex interaction of exteroceptive and interoceptive afferent streams and is significantly influenced by cognitive styles [9,10]. Two cognitive styles have been identified—field-dependent (FD) and field-independent (FI). The visual sensory system is dominant in postural control in FD subjects, whereas the dominant sensory systems in postural stabilization in FI subjects are vestibular and proprioceptive [11]. Individuals of the FD style show “en bloc” oscillations of the head–shoulder–hip unit in the absence of vision or visual stimuli [12]. Under the same conditions, in FI subjects, each body segment contributes independently to maintaining an upright posture. Thus, the activation of the flexor and extensor muscles of the legs of FD and FI subjects is likely to have different effects on the vertical posture indicators.

### 1.4. Purpose and Hypothesis of the Study

We have used a relatively new method of modulating the activity of the human SC to gain new insights into the postural stabilization of healthy subjects. We aimed to investigate the effect of tES-induced activation of the lower limb extensors on vertical postural stability in normal subjects. Our hope is that the results will be useful for clinical practice in the development of new rehabilitation of postural dysfunction.

Because postural regulation is different in subjects with different cognitive styles, the effects of stimulation on FD and FI subjects were analyzed separately. Because the effect of cognitive style on the coordination of body segments in postural control is clearly manifested in the absence of visual and sound stimuli [12,13], the study was conducted in a soundproof chamber, and participants were asked to keep their eyes closed.

Stimulation at the level of the L1–L2 vertebrae during stepping has been associated with increased coactivation of the leg muscles during the stance phase [14]. Agonist–antagonist muscle coactivation is associated with joint stiffness [15]. If tES increases muscle coactivation (joint stiffness) and joint coordination while maintaining a vertical posture differently in FD and FI subjects, then the effect of tES on vertical position will be different in FD and FI subjects.

It has been shown that in normal subjects, the active stiffness mechanisms of the joints are important for restoring the forces necessary to prevent the body from falling [16]. We hypothesized that in FI participants, where the body segments independently contribute to postural maintenance, the increase in ankle stiffness could be compensated for. In FD participants, where the upper body segments oscillate “en block” in the absence of visual and auditory stimuli, these segments are unlikely to compensate for the increase in ankle stiffness. Thus, tES may induce postural instability in FD participants and have no effect on FI participants. We did not know whether postural balance would be affected by increased joint stiffness in one leg.

## 2. Materials and Methods

The procedures and studies were performed in accordance with the tenets of the Declaration of Helsinki and approved by the Ethics Committee of the Sechenov Institute of Evolutionary Physiology and Biochemistry of the Russian Academy of Sciences (Minutes # 1-02, dated 2023/02/01). All participants gave written informed consent.

### 2.1. Participants

The study included 16 volunteers (7 males, 9 females, 25 ± 5 years, range of 18–36 years). The participants’ body mass index was 21.2 ± 2.5 kg/m^2^. All participants rated themselves as healthy on the day of the study. All participants had right leg dominance as determined by a ball kick test [17].

### 2.2. Procedure and Task

**Figure 1:**
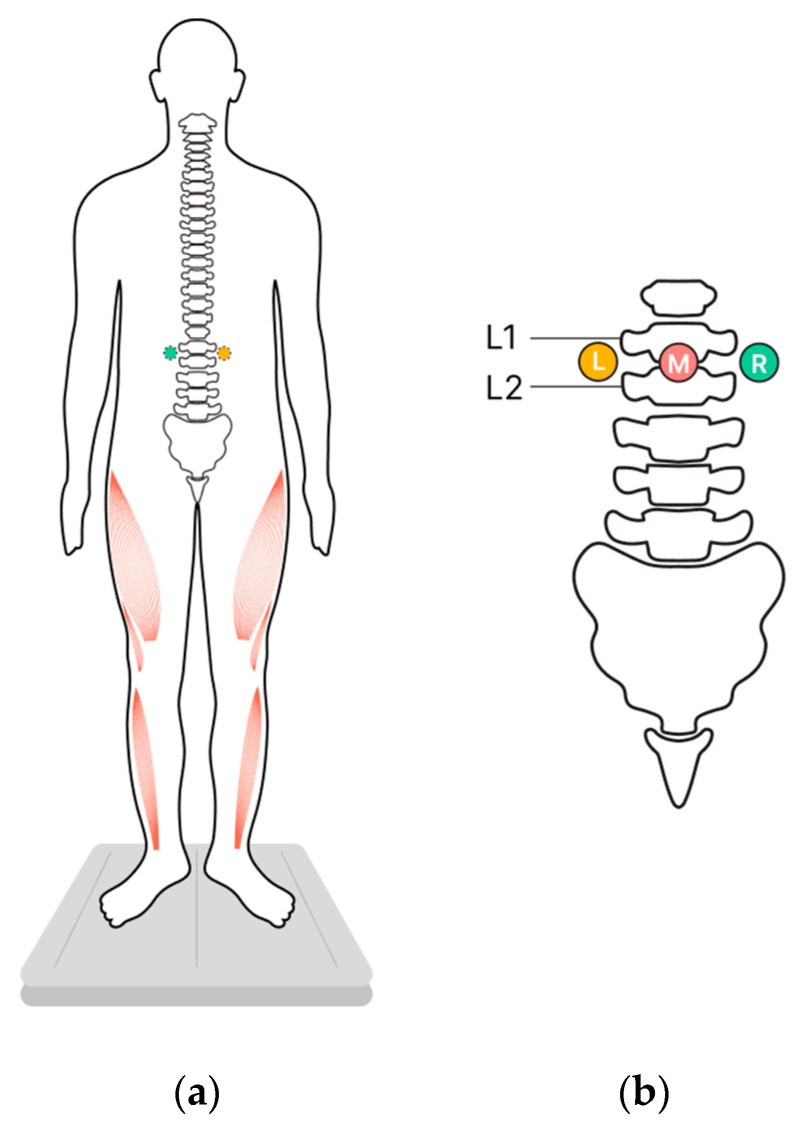
Study design. Participants stood on a force plate in a standard position while transcutaneous electrical stimulation (tES) of the spinal cord was applied to modulate extensor activity in one of three experimental conditions, or they stood in control conditions without tES. (**a**) The position of the participants, ventral view, activated extensors marked in red. (**b**) The positions of the cathodes for tES relative to the spine, dorsal view. L—left, M—midline, R—right.

Changes in vertical posture were determined by stabilometry when the participants stood on a force platform in the center of a soundproof anechoic chamber. The upright posture was standard (heels together, toes apart, hands down along the body) (Figure 1a).

The participants were instructed to maintain an upright posture with their eyes closed in four experimental conditions. Three of these conditions involved tES of the spinal cord, applied to one of these loci: in the midline between the L1 and L2 vertebrae and over the left or right dorsal roots of the spinal cord at the same level (Figure 1b). The fourth condition consisted of standing without tES, which served as the control. The position of the participant’s center of pressure (CoP) was recorded for each of the four experimental conditions.

Each recording lasted 70 s. To determine the effect of tES on posture, we analyzed the interval between 30 s and 60 s. The stimulation continued for 70 s. We excluded the preceding 30 s and the following 10 s to avoid the influence of the on-stimulation effect and the effect of waiting for the end of the recording. Breaks of 2–3 min were allowed between recordings to minimize fatigue. The participants were allowed to step off or rest on the force platform between recordings.

The order of the four recordings was randomized. After a short break (≤5 min), another random order of these four recordings followed. Each set of these four conditions was considered an independent series, as a test and a retest. Thus, we obtained two measurements for each participant in all experimental conditions.

To prevent the possibility of voluntary or unconscious effort during the tES, the participants were given a cognitive distraction task, which was to silently subtract a two-digit number from a three-digit number [7]. Mental arithmetic was also performed during the control recordings.

The cognitive style of the participants was determined using the Group Embedded Figures Test [10]. This pencil-and-paper test is the most commonly used test of field dependence and independence [18]. Those participants who scored <2.5 on the test were classified as FD, and those who scored >2.5—as FI.

### 2.3. Stabilometry

The Stabilan-01-2 force plate system (Rhythm Ltd., Taganrog, Russia) with StabMed 2.13 software was used for stabilometry [19]. The system recorded the CoP positions with a sampling frequency of 50 Hz and a resolution of <0.01 mm.

### 2.4. Transcutaneous Electrical Stimulation of the Spinal Cord and Dorsal Roots

The Neostym-5 (Cosyma Ltd., Moscow, Russia) was used for the tES. Stimulation was administered at a frequency of 20 Hz with monopolar modulated current pulses (1 ms, 5 kHz).

Adhesive cathodes (ø 2.5 cm, ValuTrode^®^ Axelgaard Manufacturing Co., Fallbrook, CA, USA) were attached to the skin of the back: one was placed along the midline between the L1 and L2 vertebrae (midline stimulation), and two were placed below and ~1.5 cm to the left and right of the midline electrode (along the dorsal roots of the spinal cord between the L1 and L2 vertebrae) (Figure 1b). Two adhesive anodes (5 × 10 cm^2^, ValuTrode^®^ Axelgaard Manufacturing Co., Fallbrook, CA, USA) were placed symmetrically above the iliac crests.

The intensity of the tES was adjusted to a maximum level that did not cause any pain or discomfort. The current intensity was selected for each tES site.

### 2.5. Analysis

#### 2.5.1. Stabilometry Indicators

The CoP parameters, including the length of the CoP trajectory along the sagittal and frontal axes, the root mean square deviation (RMSD) of the CoP along the frontal and sagittal axes, and the area of the confidence ellipse (i.e., the ellipse area), were calculated (the formulas for the calculation are in Table S1). Increased values of these dependent parameters indicate decreased postural control, while decreased parameters indicate increased postural control.

Changes in the CoP parameters between the control and experimental conditions are expressed as percentages: (experimental condition/control condition) × 100%. The absolute values of the changes in the stabilometry indicators are calculated as arithmetic differences between the parameters in the comparison conditions. A paired comparison was made between control and experimental values for each individual tested.

We also considered other parameters used in stabilometry—the average linear velocities along the frontal and sagittal axes, and shifts in the CoP along the frontal and sagittal axes. These indicators did not change significantly under any influence, so we do not describe them in the article.

#### 2.5.2. Statistics

Statistical analysis was performed using the Statistica v.10.A software package. The Shapiro–Wilk W test was used to determine whether the data followed a normal distribution. Where not all data were normally distributed, non-parametric statistics were used.

Values are presented as the mean ± standard deviation or the median (first quartile (Q1), third quartile (Q3)) depending on the data distribution.

The significance of the differences between the experimental conditions was determined using the Wilcoxon test. The significance of the differences between the parameters of the FI and FD participants was calculated using the Mann–Whitney U test.

## 3. Results

### 3.1. Cognitive Style—CoP Parameters without tES

Of the 16 participants, eight were classified as FI and eight as FD subjects. Three of the CoP parameters differed significantly between the FI and FD participants in the control condition (Table 1). In the ellipse area, the RMSDs of the CoP along the frontal and sagittal axes were ~2.5 (*p* = 0.01, Z = 2.45), 1.9 (*p* = 0.02, Z = 2.28) and 1.3 times (*p* = 0.03, Z = 2.09) higher in the FD participants than in the FI participants, respectively. These results show that the FD participants were less stable than the FI participants.

### 3.2. Current Intensities

The current intensity was adjusted individually for each participant. It was important to check whether the different values of the CoP parameters in the FD and FI participants were the result of the different tES intensities.

The current intensities for the tES ranged from 10 to 57 mA. The intensities did not differ significantly between the FD and FI participants (Table 2). The equality of the stimulation intensities in the FD and FI groups allow us to show that the differences described below were not the result of the tES intensity.

### 3.3. CoP Parameters in Experimental Conditions

**Figure 2:**
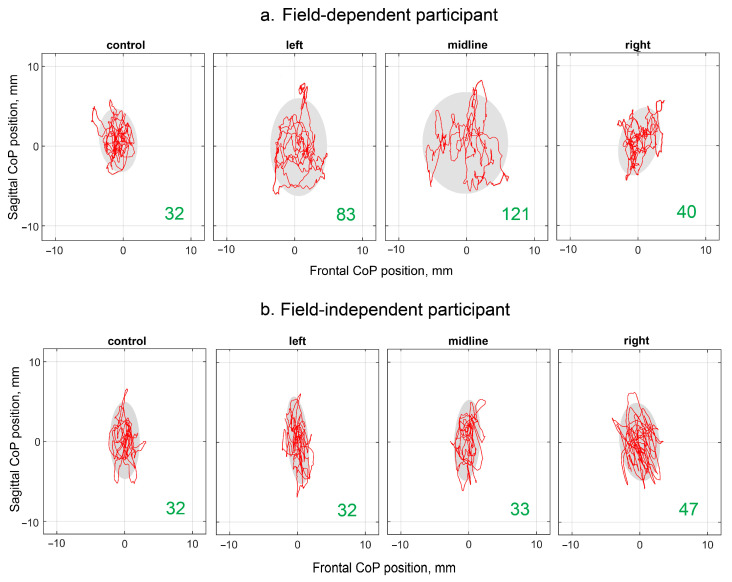
Individual CoP trajectories of the FD (**a**) and FI (**b**) participants; the period analyzed was 30 s. The plots labeled “control”, “left”, “midline”, and “right” show trajectories recorded without stimulation, and with the left, midline, and right tES, respectively. The value of the ellipse area is marked in green (mm^2^).

Examples of the CoP trajectories of the FD and FI participants in the different experimental conditions are shown in Figure 2. The individual variability of the changes in the analyzed parameters during the tES is shown in Figure S1. The increase in the ellipse area with stimulation of the left dorsal root and midline at the level of the L1 vertebra relative to the control is clearly visible in the plots of the FD participant. This effect is not present in the plots of the FI participants.

An increase in the area of the ellipse indicates a decrease in vertical balance. These primary study data show that the tES decreased the vertical stability in the FD participants and did not affect the vertical stability in the FI participants. In the FD participants, unilateral tES, which increased the stiffness of the supporting leg joints, decreased the stability. These observations were confirmed by the analysis of the changes in the indicators of the CoP trajectory induced by the SC stimulation in the groups of the FD and FI participants.

#### 3.3.1. Length of the CoP trajectory

**Figure 3:**
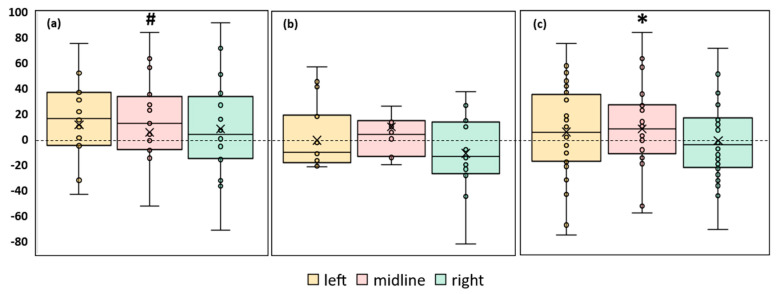
Differences between the length of the center of pressure trajectory along the frontal axis in the experimental and control conditions, in mm. The outlier points, which are either below the lower whisker line or above the upper whisker line are not shown. “Left”, “midline”, “right”—the positions of the cathodes for tES relative to the spine (Figure 1b). (**a**) FD participants; (**b**) FI participants; (**c**) all participants; * *p* < 0.05; # *p* = 0.06 compared to control.

An analysis of the CoP trajectory length along the frontal axis of the FD participants showed a tendency to increase by 23% (*p* = 0.06, Z = 1.87) during the midline tES compared to the control condition (Figure 3a, Table S2). In the FI participants, there were no significant differences between the values of this parameter in the control and stimulation conditions (Figure 3b, Table S2). The analysis of this CoP parameter in the combined group of participants revealed an increase of 8% (*p* = 0.045, Z = 1.99) during the midline tES compared to the control (Figure 3c, Table S2).

The length of the CoP trajectory along the sagittal axis in both the FI and FD participants did not show significant changes under the stimulation conditions compared to the control. A similar result was obtained in the combined group.

In conclusion, the analysis of the length of the CoP in the mixed group shows a significant decrease in the balance stability during the midline spinal stimulation. According to this parameter, the balance stability of the FD participants had a tendency to decrease and was not affected by the stimulation in the FI participants.

#### 3.3.2. Ellipse Area

**Figure 4:**
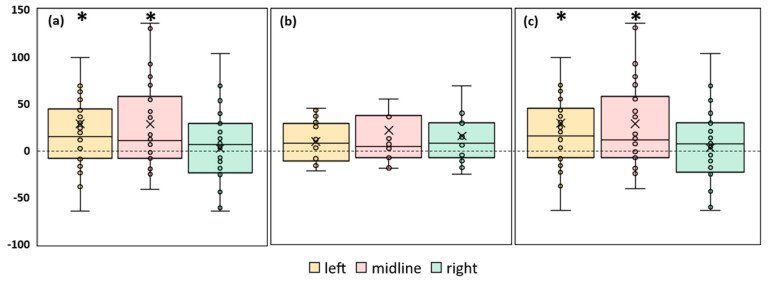
Differences in ellipse area between experimental and control conditions, in mm^2^. The outlier points that lie either below the lower whisker line or above the upper whisker line are not shown. “Left”, “midline”, “right”—the positions of the cathodes for tES relative to the spine (Figure 1b). (**a**) FD participants; (**b**) FI participants; (**c**) all participants; * *p* < 0.05 compared to control.

A significant increase in the ellipse area during stimulation was observed in the FD participants. The left roots and midline tES increased the ellipse area by ~30% (*p* = 0.03, Z = 2.17 and *p* = 0.046, Z = 1.99, respectively) (Figure 4a, Table S3). No significant changes in the ellipse area were observed in the FI participants (Figure 4b, Table S3). In the combined group, this CoP parameter significantly increased by 23% during tES of the left root (*p* = 0.01, Z = 2.56) and by 27% during midline stimulation (*p* = 0.02, Z = 2.29) (Figure 4c, Table S3).

Thus, according to the ellipse area parameter, the balance decreased in the combined group and in the FD subgroup for the midline and left root spinal stimulation. Spinal stimulation did not affect the equilibrium of the FI subgroup.

#### 3.3.3. RMSD of the CoP

**Figure 5:**
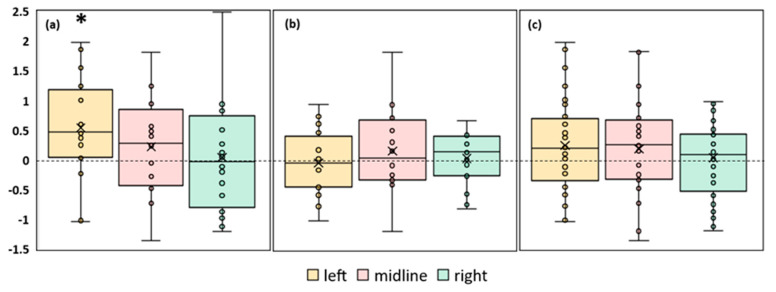
Differences between the root mean square deviation of the center of pressure along the frontal axis in the experimental and control conditions, in mm. The outlier points that lie either below the lower whisker line or above the upper whisker line are not shown. “Left”, “midline”, “right”—the positions of the cathodes for tES relative to the spine (Figure 1b). (**a**) FD participants; (**b**) FI participants; (**c**) all participants; * *p* < 0.05 compared to control.

A significant 18% increase in the RMSD of the CoP along the frontal axis (*p* = 0.04, Z = 2.02) was observed in the FD participants during the tES of the left roots (Figure 5a, Table S4). No significant changes in this parameter were observed during the stimulation in the FI participants or in the combined group (Figure 5b,c, Table S4).

**Figure 6:**
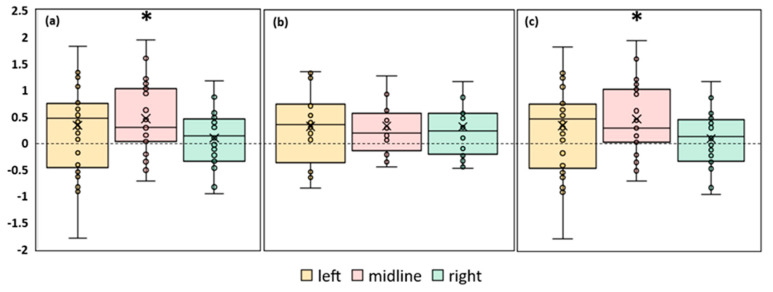
Differences in root mean square deviation of the center of pressure along the sagittal axis between experimental and control conditions, in mm. “Left”, “midline”, “right”—the positions of the cathodes for tES relative to the spine (Figure 1b). (**a**) FD participants; (**b**) FI participants; (**c**) all participants; * *p* < 0.05 compared to control.

The RMSD of the CoP along the sagittal axis increased by 15% (*p* = 0.02, Z = 2.33) during the midline tES in the FD participants (Figure 6a, Table S5). There were no significant changes in this RMSD of the CoP in the FI participants (Figure 6b, Table S5). In the combined group, this parameter increased by 12% (*p* = 0.008, Z = 2.64) during the midline tES (Figure 6c, Table S5).

According to the RMSD of the CoP along the sagittal axis, tES at the midline electrode position increased instability in the FD subgroup and the combined group. According to the RMSD of the CoP along the frontal axis, the stimulation of the left spinal roots destabilized the balance of the FD participants.

#### 3.3.4. Differences in CoP Parameters between Left and Right tES

**Figure 7:**
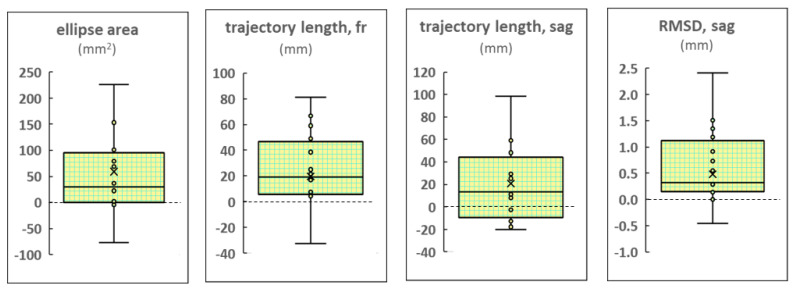
Differences in the center of pressure parameters obtained during stimulation of the left and right roots of the spinal cord. Results of the FD participants. RMSD: the root mean square deviation.

In the FD participants, most of the CoP parameters obtained during the tES of the left roots were significantly greater than those obtained during the stimulation of the right root. The differences in these parameters are shown in Figure 7. The other indices did not differ significantly. The ellipse area was greater by 37% (*p* = 0.02, Z = 2.38), the RMSD of the CoP along the sagittal axis—by 13% (*p* = 0.03, Z = 2.21), the trajectory length along the frontal axis—by 13% (*p* = 0.03, Z = 2.22) (Tables S3, S5, and S2, respectively), and the trajectory length along the sagittal axis by 10% (*p* = 0.04, Z = 2.07). The elevated values of these dependent parameters indicate decreased postural control. This effect was absent in the FI participants and in the combined group.

## 4. Discussion

### 4.1. Differences in Postural Control Responses to Spinal Test of Patients with Motor Disorders and Healthy Subjects

Previously, electrical stimulation of the cervical and lumbar segments of the spinal cord combined with multi-session activity-based training has been shown to rehabilitate voluntary movements and independent body weight support in patients with severe spinal cord injury [5,20,21]. Acute spinal tES improved postural stability in 11 of 12 patients (aged 2–50 years) with cerebral palsy [22]. In subjects with multiple sclerosis, postural stability was improved during tES of the spinal cord when standing with eyes closed, presumably by modulating proprioceptive function [23]. Research on laboratory animals has shown that spinal cord stimulation activates antigravity tonic muscles and restores spinal networks that control body balance [2,24].

The effect of spinal tES on balance control was studied in healthy adults [25,26]. The tests included forward, backward, and left and right trunk perturbations with and without midline stimulation between the L2 and L3 vertebrae. The disturbances were caused by forces applied to the pelvis. Continuous monophasic tES consisted of pulses of 5–10 mA, at a frequency of 30 Hz. Balance control was characterized based on the participants’ responses to the force perturbations. It was found that the acute effect of tES was to increase muscle activity during the forward, left, and right perturbations, but this was accompanied by reduced balance performance in the forward direction.

Thus, an increase in muscle strength during tES in patients is followed by an increase in vertical stability, and in healthy subjects, vertical stability decreases with the simultaneous increase in muscle strength caused by stimulation.

We investigated the effect of tES on postural control when healthy adult subjects maintained steady-state upright postures. Midline tES reduced the postural stability, as evidenced by an increase in the CoP trajectory length along the frontal axis, confidence ellipse area, and RMSD of the CoP along the sagittal axis during midline stimulation (Figure 3c, Figure 4c, and Figure 6c, respectively).

Therefore, spinal tES at the L1–L2 vertebral level activates the lower limb extensors [6]. This stimulation induces and restores independent upright posture in patients with motor disorders, whereas it decreases postural stability in healthy subjects. The reasons for these different effects can be speculated. In the patients, tES of the balance networks of the spinal cord, in addition to increasing muscle strength, replaces the missing or malfunctioning supraspinal balance control. Thus, the patients’ independent upright posture is rehabilitated during stimulation. In healthy subjects, tES may disrupt the normally functioning supraspinal regulation of vertical posture, and antigravitational stability decreases under this effect. This assumption is supported by the different results of tES on upright posture in FD and FI participants.

### 4.2. Upright Posture of FD Participants Responded to Spinal tES and That of FI Participants Did Not

In 1948, the rod and frame test, in which a rod and outer frame are rotated independently at variable angles, was introduced to assess subjective vertical position in space [27]. Subjects were divided into two groups, each referred to as a particular cognitive style, based on their response to the test. Subjects who oriented the rod to tilt in the direction of the tilted frame were identified as field-dependent (FD) because they relied on the visual field defined by the frame to estimate verticality. Later, it was shown that not only the visual system but multiple sensory systems are involved in generating an internal representation of the body in space, perceiving one’s own movement, and maintaining upright posture [28].

Isableu et al. showed that cognitive style has a significant impact on one’s postural control strategy [11]. Different efficiencies in postural performance were exhibited by FD and FI subjects—the FD subjects were less stable than the FI subjects. Similarly, in our study (Table 1), the increased values of the ellipse area, RMSD_front_, and RMSD_sag_ in the FD participants compared to the FI participants indicate decreased postural control in the FD participants in control conditions.

Strategies of the segmental coordination of the head, shoulders, and hips during upright standing were studied in the FD and FI subjects [12]. Segmental stabilization strategies differed among these individuals. FD subjects showed increased hip stabilization, and this stabilization was associated with “en bloc” operation of the head/shoulder and/or shoulder/hip units, which induced corresponding stability of the entire trunk and head. In the FI subjects, the head, shoulders, and hips moved independently from each other during postural control.

The relationship between joint stabilization and upright postural instability has been demonstrated [29]. The young adults stood on a force plate for 60 s without and with immobilized joints (only knees constrained, knees and hips, and knees, hips, and trunk). It was shown that increased body stability was achieved when more joints were free to move.

As we hypothesized, tES decreased upright postural stability in the FD participants but not in the FI participants (Figure 2, Figure 3, Figure 4, Figure 5 and Figure 6). The intensity of the stimulation current was the same in the subgroups (Table 2), and thus, the different effects of the tES on the upright posture of the FD and FI participants are related to the different strategies for body control in these subjects. It has been shown that tES at the level of the L1 vertebra during the stance phase of stepping increases the coactivation of the antagonist muscles [14]. The coactivation index values increased by ~10% for thigh muscles and ~5% for ankle muscles. Increased agonist–antagonist muscle coactivation results in increased apparent joint stiffness [15].

In FI subjects, body segments move independently from each other during steady-state standing [12]. The increased stiffness of the ankle and knee joints induced by tES could be compensated by the increased flexion of the other segments during postural control, which is why the recorded postural parameters were not altered significantly in these participants.

In FD subjects, body segments are “en bloc” during postural control [12]. The increased stiffness of the leg joints during tES increased the overall stabilization of the joints, and the instability of the upright posture was similar to that previously demonstrated when the joints were mechanically stabilized [29].

Thus, tES decreased postural stability in the FD participants and had no effect on the FI participants. Cognitive style is important in tES studies of postural control in normal subjects.

### 4.3. Left and Right Spinal Root tES Had Different Effects on Postural Balance

Computational modeling of spinal tES showed that midline spinal stimulation increased the current density around the spinal segments but was clearly concentrated along the dorsal roots on both sides, and the lumbar lateral cathode stimulated the vast majority of the lumbar nerve roots on the side of stimulation [30].

The effects of tES of the left and right spinal roots differed in the FD subgroup as well as in the combined group. Stimulation of the left spinal root significantly decreased postural control, whereas stimulation of the right spinal root did not (Figure 4 and Figure 5). In the FD participants, the values of four CoP parameters during the tES of the left root were significantly higher than the values of these parameters during the stimulation of the right root (Figure 7). The results show that postural stability during the tES of the left root was less than that without the stimulation of the right root.

All participants in our study had right leg dominance, so their left leg was the supporting leg [17]. Thus, the tES of the dorsal roots at the L1 vertebra on the side of the supporting leg reduced postural control, and the one on the side of the dominant leg did not.

Based on a recent meta-analysis that reviewed a large number of experimental studies to answer the question of whether leg dominance affected upright postural stability in healthy adults, it was concluded that balance performance is not influenced by leg dominance [31]. It should be noted that all studies included in this review used a one-foot balance test in one form or another. We were unable to find any studies using a bipedal stance that investigated the role of the dominant or supporting leg in maintaining an upright posture. Therefore, future studies are needed to interpret the fact that precise stimulation of the left/right spinal root at the level of the L1 vertebra has different effects on postural balance.

### 4.4. “Posture” and “Equilibrium” Tasks during Standing and Spinal tES

As mentioned above, the control of balance during standing solves two tasks simultaneously: one is the stabilization of the body segments to resist gravity (“posture”), and the other is the compensation of perturbations of the body segments (“equilibrium”) [7]. The “posture” task is mainly performed by the extensor muscles, and the “equilibrium” task is mainly performed by the flexor muscles. Spinal stimulation at L1–L2 mainly activates the extensors, and at T11–T12, mainly the flexors [6].

We have shown that tES of the spinal cord at the L1–L2 vertebrae reduces postural control during quiet standing when the “equilibrium” task is virtually absent. Omofuma and colleagues [25,26] stimulated a similar level of the spinal cord and tested balance control during trunk perturbations when the “equilibrium” task was solved simultaneously with the “posture” task. They found that balance suffered. In both studies, the tES interfered with the control of the antigravity tonic muscles. As a next step, we plan to study the effects of stimulation at the T11–T12 vertebrae to activate the flexor muscles during vertical postural perturbations.

### 4.5. Limitations

We were not able to use electromyography and motion capture methods together with stabilometry due to the limitations of using a soundproof anechoic chamber. This would have allowed us to confirm the increase in joint stiffness during stimulation at the level of the L1 vertebra and to analyze the actual involvement of the body segments in maintaining the upright posture by the participants.

## 5. Conclusions

Spinal tES at the L1–L2 vertebrae, used for the neurorehabilitation of standing in patients with motor disorders, reduced balance control in healthy participants.

Cognitive style is important in postural control. The separate analyses of the results of the FD and FI subgroups showed that tES did not affect postural control in the FI subjects.

We attribute this effect to an increase in lower limb joint stiffness, which is critical for postural control in FD subjects.

The tES of the dorsal roots at the L1 vertebra on the side of the supporting leg reduces postural control, and the one on the side of the dominant leg does not.

This non-invasive spinal cord stimulation can be used to study the regulatory mechanisms of upright standing in healthy subjects. New knowledge can be used to develop rehabilitation methods for postural disorders.

## Figures and Tables

**Table 1:** Postural parameters of the FI and FD participants. *N* is the number of recorded episodes. Tests and retests were considered for each participant.

Participants	Ellipse Area, mm^2^	RMSD_front_, mm	RMSD_sag_, mm	
FD (*N* = 16)	151 (86; 231) *	3.1 (2.0; 3.7) *	3.1 (2.3; 4.0) *	
FI (*N* = 16)	56 (46; 102)	1.6 (1.6; 2.6)	2.4 (2.1; 2.7)	
All (*N* = 32)	93 (50; 148)	2.2 (1.6; 3.2)	2.6 (2.1; 3.3)

RMSD_front_—root mean square deviation of the center of pressure along the frontal axis; RMSD_sag_—root mean square deviation of the center of pressure along the sagittal axis; * *p* < 0.05 relative to the results of the FI participants.

**Table 2:** Current intensities of the tES. Stimulation was applied between the L1 and L2 vertebrae (“midline”) and to the left and right of the midline electrode (“left dorsal roots” and “right dorsal roots”, respectively). Number of participants displayed (*N*).

Participants	Left Dorsal Roots, mA	Midline, mA	Right Dorsal Roots, mA
FD (*N* = 8)	21 ± 6	26 ± 13	21 ± 6
FI (*N* = 8)	20 ± 11	22 ± 12	19 ± 10
All (*N* = 16)	20 ± 11	24 ± 12	20 ± 10

## Data Availability

The datasets generated during and/or analyzed during the current study are available from the corresponding author upon reasonable request.

## Supplementary Materials

The following supporting information can be downloaded at: https://www.mdpi.com/article/10.3390/life13091909/s1, Figure S1: Individual variability of changes in analyzed parameters during stimulation; Table S1: Analyzed center of pressure (CoP) parameters; Table S2: Length of the CoP trajectory along the frontal axis based on participant’s cognitive type and tES condition (mm); Table S3: Ellipse area based on participant’s cognitive type and tES condition (mm^2^); Table S4: RMSD along the frontal axis based on participant’s cognitive type and tES condition (mm); Table S5: RMSD of CoP along the sagittal axis based on participant’s cognitive type and tES condition (mm).
