# Supplementary material for: Posture of Healthy Subjects Modulated by Transcutaneous Spinal Cord Stimulation
Source: Life (Basel). 2023 Sep 14;13(9):1909. doi: 10.3390/life13091909 (PMC10532446; doi:10.3390/life13091909)
Supplement: Supplementary file 1 [file life-13-01909-s001.zip › life-2574944-supplementary.pdf]

Table S1. Analysed center of pressure (CoP) parameters

| Parameter | Definition | Formula |
|---|---|---|
| Length of the CoP trajectory along the frontal axis | Length of the frontal component of the CoP signal | $LX = \sum_{i=1}^{N} (X_i - X_{i-1})$ |
| Length of the CoP trajectory along the sagittal axis | Length of the sagittal component of the CoP signal | $LY = \sum_{i=1}^{N} (Y_i - Y_{i-1})$ |
| RMSD along the frontal axis | Root mean square deviation of the CoP position along the frontal axis | $Q_x = \sqrt{\frac{1}{N-1} \sum_{i=1}^{N} \left(X_i - X_{cp}\right)^2}$ |
| RMSD along the sagittal axis | Root mean square deviation of the CoP position along the sagittal axis | $Q_y = \sqrt{\frac{1}{N-1} \sum_{i=1}^{N} \left(Y_i - Y_{cp}\right)^2}$ |
| Confidence ellipse area | The main part of the area occupied by the CoP without so-called loops and accidental outliers | $S_{\text{yëë}} = 2 \ln \frac{1}{1-\beta} \sqrt{D(X) \cdot D(Y) - Cov(X,Y)^2}$ |

$X_i$. $Y_i$ – CoP coordinates in time

N – number of counts

β –probability that the point of the statokinesiogram hits into the ellipse (β = 0.9).

D(X), D(Y) – corresponding component dispersion

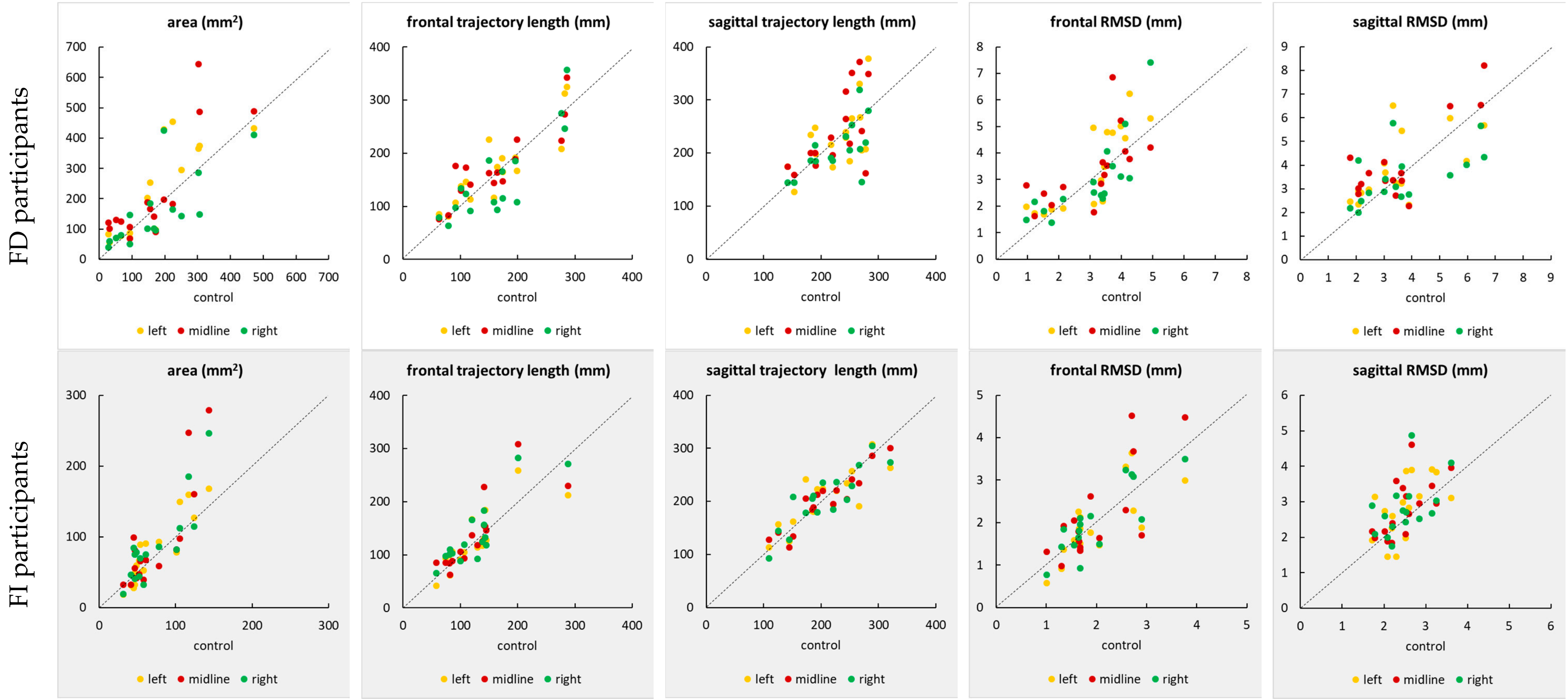


Figure S1. Individual variability of changes in the analyzed parameters during stimulation. Data from eight field-dependent (FD) and eight field-independent (FI) participants are shown. Four recordings: "control", "left", "midline" and "right" - without stimulation, with stimulation of the left dorsal roots, midline and right dorsal roots, respectively. The order of the four recordings was randomized during each test of a participant. After a short break, another random order of these four recordings followed. Each set of these four conditions was considered an independent series, a test and a retest. Thus, we obtained two measurements for each participant in all experimental conditions. For each participant there are six points on the plot: two of each color, representing test and retest in each of the three conditions.

Table S2. Length of the CoP trajectory along the frontal axis based on participant’s cognitive type and tES condition (mm).

| participants | Control | left tES | midline tES | right tES |
|---|---|---|---|---|
| FD (N=16) | 155 (107; 196) | 165 (114; 195)[%] | 164 (142; 205)[#] | 119 (96; 185) |
| FI (N=16) | 126 (85; 142) | 116 (98; 139) | 121 (87; 153) | 119 (100; 158) |
| All (N=32) | 135 (98; 166) | 129 (105; 186) | 146 (99; 182)[*] | 119 (97; 169) |

*p = 0.04, Z = 1.99, #p = 0.06, Z = 1.87 compared to control condition;

[%]p = 0.03, Z = 2.22 compared to right tES

Table S3. Ellipse area based on participant’s cognitive type and tES condition ($mm^2$)

| participants | Control | left tES | midline tES | right tES |
|---|---|---|---|---|
| FD (N=16) | 151 (86; 231) | 153 (81; 367)[*, %] | 142 (113; 192)[#] | 122 (77; 169) |
| FI (N=16) | 56 (46; 102) | 73 (44; 101) | 66 (45; 98) | 76 (45; 92) |
| All (N=32) | 93 (50; 148) | 90 (65; 176)[$] | 101 (66; 174)[£] | 85 (67; 147) |

*p = 0.02, Z = 2.17; [#]p = 0.04, Z = 1.98 ; [$]p = 0.01, Z = 2.56; [£]p = 0.02, Z = 2.29 compared to control condition;

[%]p = 0.02, Z = 2.38 compared to right tES

Table S4. RMSD of CoP along the frontal axis based on participant’s cognitive type and tES condition (mm). RMSD: the root mean square deviation.

| participants | Control | left tES | midline tES | right tES |
|---|---|---|---|---|
| FD (N=16) | 3.1 (2.0 ;3.7) | 3.2 (1.9; 4.8)[*] | 3.1 (2.5; 3.9) | 2.4 (2.2; 3.2) |
| FI (N=16) | 1.6 (1.6; 2.6) | 1.8 (1.5; 2.2) | 1.7 (1.4; 2.3) | 1.9 (1.4; 2.3) |
| All (N=32) | 2.2 (1.6; 3.2) | 2.1 (1.7; 3.5) | 2.4 (1,6; 3.6) | 2.2 (1.7; 3.0) |

*p = 0.02, Z = 2.22 compared to control condition

Table S5. RMSD of CoP along the sagittal axis based on participant's cognitive type and tES condition (mm). RMSD: the root mean square deviation.

| participants | control | left tES | midline tES | right tES |
|---|---|---|---|---|
| FD (N=16) | 3.1 (2.3; 4.0) | 3.4 (2.8 ;5.5) $^{\%}$ | 3.3 (3.1; 4.2)$^{*}$ | 3.2 (2.7; 4.0) |
| FI (N=16) | 2.4 (2.1; 2.7) | 2.9 (2.1; 3.3) | 2.8 (2.1; 3.3) | 2.6 (2.3; 3.0) |
| All (N=32) | 2.6 (2.1; 3.3) | 3.1 (2.5; 3.9) | 3.2 (2.5; 3.6)$^{\#}$ | 2.8 (2.5; 3.6) |

*p = 0.01, Z = 2.32; #p = 0.004, Z = 2.84 compared to control condition

$^{\%}$p = 0.03, Z = 2.21 compared to right tES
